# Unethical conduct as a multifaceted phenomenon in psychiatric care: Nurse leaders’ perspectives

**DOI:** 10.1177/09697330241299523

**Published:** 2024-11-13

**Authors:** Julia Björklund, Jessica Hemberg

**Affiliations:** Åbo Akademi University; Åbo Akademi University

**Keywords:** Perceptions, psychiatric care, nurse leaders, unethical conduct

## Abstract

**Background:** Mental healthcare can be considered a unique practice due to its ethical characteristics, and an awareness of ethics is crucial when working in a mental health setting. Several ethical challenges exist, and professionals may not always recognize the ethical aspects of psychiatric care. Research on psychiatric care from nurse leaders’ perspective is scarce but important, because nurse leaders can impact and cultivate workplace culture. **Aim:** To explore the phenomenon of unethical conduct in a psychiatric inpatient context from nurse leaders’ perspectives. **Research design:** Qualitative exploratory design. In-depth semi-structured interviews. **Participants and research context:** Eight nurse leaders from two different healthcare organizations in Finland. Leadership experience ranged between 2 and 30 years. **Ethical considerations:** Research ethics permission was received from a Research Ethics Board where the researchers are domiciled. Guidelines on good scientific practice as delineated by the Finnish Advisory Board on Research Integrity TENK were followed. **Findings:** Six main categories were generated: Unethical conduct and violations against patients, Unethical conduct and violations against staff, Unethical conduct and violations by staff against other staff, Unethical conduct and violations against leaders, Reasons underlying unethical conduct, and Consequences of unethical conduct and the positive development of psychiatric care. **Conclusions:** Unethical conduct was seen to be a multifaceted phenomenon, and patients and staff alike can experience and engage in unethical conduct. Unethical conduct against patients was linked to power imbalance (nature of involuntary care, staff attitudes) and a focus on rules based in historical precedent (paternalistic, routine-focused, not patient-centered). Unethical conduct against staff was linked to the nature of involuntary care and patient ill-health. Unethical conduct by staff against other staff was linked to a lack of understanding for others’ work, interpersonal chemistry, (length of) work experience, and staff character. Unethical conduct against leaders was linked to leaders being perceived as the organization.

## Introduction

Mental healthcare can be considered a unique practice due to its ethical characteristics^1^ and an awareness of ethics is crucial when working with patients in all contexts, since all patients can be seen as vulnerable. To promote patient well-being in mental healthcare, nurses should incorporate a focus on human rights and ethics when providing care in this context.^2^

In research on ethics in mental health care and psychiatric care, the phenomenon of coercion and its umbrella terms are frequently explored. For example, researchers have previously studied coercion, informal coercion,^1,3,4^ dignity,^5^ patient autonomy, paternalism toward patients,^1^ the patient encounter, and the nurse–patient relationship.^6,7^ Also, nurses’ experiences and ethical conduct when encountering patient violence in psychiatric care have been investigated.^8^

Within a medical context, the word “ethical” can be defined as *conforming to accepted professional standards of conduct*.^9^ Unethical conduct can be defined as *any act involving the deliberate violation of accepted or agreed ethical standards* while unethical professional conduct can be defined as conduct that is *contrary to the accepted standards of a profession*.^10^ Research suggests that, despite being of upmost importance, the topic of professional ethics has not been thoroughly researched within the discipline of nursing science.^11^

As seen in the International Council of Nurses’ (ICN) code of ethics,^12^ nursing should include a respect for basic human rights and patients should be treated with dignity and respect. The nursing theorist Katie Eriksson has described the inner core of caring, that is, the ethos of caring, as comprising compassion and love toward a fellow human being.^13^ However, in accordance with the theory of suffering related to care, healthcare systems can even at times cause patients further suffering.^14^

## Background

The World Health Organization (WHO) defines mental health as being a basic human right and considers it to be a more multifaceted phenomenon than the absence of mental ill-health.^15^ In the WHO definition, mental health is perceived as existing on a complex continuum; it is a state of being that allows individuals to manage life stresses, learn, realize their abilities, and contribute to their communities.^15^

In Finland, the stated goal of mental health work is the strengthening of individuals’ good mental health.^16^ Such work can encompass activities related to the prevention, alleviation, and treatment of mental health disorders, for example, guidance, psychosocial support, support during crisis, or rehabilitation.^16^ Per legislation, mental health services are primarily organized as out-patient care and through health centers.^17^ About 21, 000 people are hospitalized annually in Finland because of mental ill-health, with one-third of such patients being involuntarily treated under the Mental Health Act.^17^

Involuntary treatment can expose patients to coercive care interventions.^18^ Overseen by local authorities in Finland, involuntary care is regulated through the Mental Health Act. This act includes the provision that the opinions of those being involuntarily treated shall be heard and defines the general conditions for the eventual limitation of a person’s fundamental rights.^19^ While researchers have recently found that psychiatric care and mental health services in Finland are of high quality, they have also identified some areas where improvement would be needed, for example, patients’ right to exercise legal capacity and personal liberty, and patients’ security.^20^ Other researchers have even identified the existence of ethical issues in psychiatric care in Finland in studies including former patients.^21^

In a study from Portugal, researchers have found that patients in in-patient hospital psychiatric settings can experience adverse events and victimization during their hospitalization.^22^ A study from the United States of America showed that involuntary care periods can erode subsequent trust in care providers and future help-seeking and that some patients can even warn peers to not seek help from certain institutions.^23^ Simha and Pandey^24^ found that nurse managers and leaders should try and establish principled and ethical climates in order to engender trust in the organization. Ethical leadership is key resource for making employees feel fair, just, and affirmed by the organization, thus further enhancing their motivation and creativity.^25^

Research on various forms of mental healthcare and psychiatric care has been conducted from the perspective of patients^26,27,3,28,29^ and staff members.^30–33,4^ Research on psychiatric care from nurse leaders’ perspective is scarce but important,^31^ because nurse leaders can impact and cultivate workplace culture.^33,34^

Coercion, and especially the ethical justification of coercion, is a frequent focus of research and comment in the study of ethics in a psychiatric context. The WHO defines coercion as *any action or practice undertaken which is inconsistent with the wishes of the person in question*.^35^ Coercive interventions can also be defined as those practices in psychiatric care that are non-consensual or forced upon those in involuntary care.^36^ Informal coercion can be defined as interventions that prohibit patients’ autonomy and decision-making, for example, persuasion, interpersonal leverage, inducement, or even threats.^37^ Healthcare staff can justify their use of informal coercion, perceiving that they are trying to do what is best for the patient or are concerned that the patient is not receiving adequate treatment.^4^

Per legislation in Finland, individuals’ fundamental rights and right to self-determination can be limited during involuntary care,^19^ which can be seen as a type of formal coercion. For example, measures such as seclusion; physical or mechanical restraint; treatment, examination, or medication regardless of patient’s will; the limitation of freedom of movement; checking and seizure of personal property; frisking and bodily search; or limitation of contacts can be undertaken.^19^

Despite a lack of scientific evidence supporting the benefit of such, coercive interventions are largely considered to be acceptable in psychiatric care.^1^ To reduce violence and the use of coercion in psychiatric wards, various interventions have been developed, for example, the Safewards Model.^38,39^ Nevertheless, several ethical challenges exist and mental health professionals may not always recognize the ethical aspects of psychiatric care.^32^ Coercive interventions can be overused in psychiatric care and are not always used as a last resort.^26,21^ Questionable practices can be used, for example, the use of excessive force, paternalistic attitudes, a lack of empathy, or a lack of communication and interaction.^26^

Patients can desire more communication and presence from staff when coercive interventions are implemented.^40^ Patients seek empathy, respect, and understanding from staff and desire that staff communicate efficiently and believe in recovery.^7^ Patients can also perceive that staff can at times lack empathy, with patients experiencing staff as being insensitive, unsupportive, or unresponsive.^27^ According to the theory of suffering related to care, care can cause patients further suffering.^14^

Also care staff can experience that patients can act in an unethical manner. For example, patients in social and healthcare settings can engage in verbal violence, threats, physical violence, or aggression toward staff, with such being a significant problem worldwide.^41,8,42^ According to WHO, up to 38% of healthcare workers may experience physical violence at some point during their careers.^45^ Workplace violence can have a significant effect on social and healthcare workers’ well-being at work and can impact their commitment and work performance.^43^ Mental health staff suffer in a larger extent from poor well-being and burnout compared to staff in other healthcare sectors. This can reflect negatively on patient safety and the quality of care.^44^

Still, workplace violence can take many forms, and worker-to-worker violence and workplace bullying can also negatively impact healthcare workers’ well-being at work.^45–47^ In a systematic review on “upwards violence” in nursing, that is, mobbing or bullying conducted by nurses against, for example, nurse managers, researchers have seen that the physical and psychological health of those leaders who are the target of such behavior can be negatively impacted.^48^ Nurses can experience mobbing from subordinates (16%), administrators (36%), or co-workers (48%).^49^

Nurse leadership is important in creating an ethical climate in the workplace.^50,51,34,52^ Nurse leaders can be considered responsible for the realization of evidence-based and good quality care, for their staff and staff well-being, and for creating a caring culture that is built on an ethical foundation.^51,52^ By fostering and promoting a culture of ethical sensitivity, leaders can influence staff to uphold high ethical standards. In caring administration, according to Bondas,^53^ the aim is to foster a workplace culture where an ethos of caring is made visible. The leader carries a responsibility for what occurs in the workplace and is expected to intervene if violations such as noncaring or uncaring take place.^53^ Research shows that ethical leadership correlates with ethical climate and with organizational citizenship behavior in healthcare organizations.^54^

In a systematic literature review research on ethical challenges linked to the use of coercion in mental healthcare is lacking and suggest further research on the topic in different clinical contexts and from varying perspectives, including a leadership perspective^l^. More research on coercion in mental healthcare from a leadership perspective should be undertaken.^33^ Research on professional ethics is scarce, noting that because professional ethics is fundamental to nursing and that not engaging in further research may cause ethics in nursing to become static it is highly important to update research on this topic.^11^ Emanating from the gap in the current body of research, the focus of this study was the phenomenon of unethical conduct in a psychiatric context as explored from a nurse leadership perspective, since the role of leadership in creating ethically sound work environments is seen as crucial for caregivers’ well-being and safety which in turn affects patients.

## Aim

The aim of the study was to explore the phenomenon of unethical conduct in a psychiatric in-patient context from nurse leaders’ perspectives.

### Theoretical perspective

The theoretical perspective used in this study is the theory of caritative caring^14^ since this theory can shed light on the importance of safeguarding patients’ dignity and alleviating suffering as well as displaying how an ethical tone set by leaders can promote staff well-being and safety which in turn can affect patients. In accordance with the caritative caring theory, the purpose of caring is to eliminate human suffering. However, because suffering cannot be wholly eliminated, healthcare professionals should instead strive to alleviate suffering in the caring encounter.^14^

Although the fundamental purpose of healthcare systems and healthcare organizations is to alleviate suffering, healthcare systems can even sometimes cause patients suffering. Suffering can be related to life, illness, or care, and suffering related to care should be eliminated because it violates the patient’s dignity and autonomy. To (re)gain good health and recover, the patient must experience that they are valued as a human being and met with dignity by being seen and confirmed.^14^

A lack of caritative caring can result in suffering for the patient, which also encompasses the absence of care, non-care, and/or healthcare professionals’ abuse of power. The absence of care can be defined as when healthcare professionals fail to see the patient’s true needs, resulting in the patient not receiving the correct care. Non-care can be defined as the lack of the caring aspect in the caring encounter. Furthermore, if healthcare professionals use their authority to influence a patient to act in a way that the patient does not wish to, this can be considered an abuse of power, which violates the patient’s dignity.^14^

A culture where the patient feels welcome and respected as a human being is needed to minimize the suffering that can be caused by healthcare. Alleviating the patient’s suffering first and foremost entails not violating the patient’s dignity, not judging or punishing the patient, and ensuring that the patient does not experience healthcare professionals’ abuse of power in the caring encounter. By mediating hope, presence, openness, and compassion, healthcare professionals can truly be present and see each unique patient and their needs, thereby minimizing experiences of suffering.^14^

The theory of caritative leadership describes a nurse leader’s main responsibility as cultivating a care culture through compassion and ethical responsibility. The theory combines caring and administration with a purpose of ministering to the patients. The leader presents ethical sensitivity and compassion, and any behavior compromising human dignity is unacceptable. The theory highlights the importance of open dialogue among healthcare teams, including non-professionals, to ensure that the core mission of ministering to patients is maintained. Caritative leadership promotes ethical decision-making and fostering a care culture where the ethos of caring is visible.^53^ Solbacken et al.^55^ describe the relationship between caring for staff and caring for patients as two sides of the same coin. Further, when leaders experience isolation in their roles, they may adopt a spectator mentality that does not nurture a caritative caring culture.^53^

## Methodological aspects

A qualitative exploratory design was used. Data were collected using in-depth semi-structured interviews.

### Data material, collection, and analysis

Contact persons at two healthcare organizations in Finland sent an email to selected nurse leaders regarding participation in the study and containing information about how the data would be used and participants’ right to withdraw from the study without further explanation. Of those contacted, nine nurse leaders indicated that they would like to participate in the study. The recruitment of participants took place during the COVID-19 pandemic in the spring of 2022 and during a strike among nurses in Finland. This period was a challenging time to recruit nurse leaders to participate in the study. One participant cancelled their participance due to more urgent matters. Participants worked at the time as first-line nurse managers (ward managers and assistant ward managers). The assistant ward managers participated in clinical work in addition to leadership tasks. The inclusion criterion was a minimum of 1 to 2 years of leadership experience in an in-patient psychiatric context. In total, eight nurse leaders were included. The participants were aged between 32 and 60 at the time of their interviews and their leadership experience ranged between 2 and 30 years.

Data collection occurred using semi-structured interviews conducted in either the Finnish or Swedish language, in accordance with participant preference. Interviews were conducted either face-to-face or online through videography in the spring of 2022. The interviews lasted between 43–79 min and were recorded and transcribed verbatim. Questions included in the interview guide were, for example: What kinds of different ethical challenges arise in a psychiatric ward? Can you give an example and tell about an incident involving ethical challenges? In your opinion, what are the risks for patients potentially being subjected to unethical behavior? Which different aspects are important to consider as a caregiver in psychiatric care and in interacting with the patient to avoid unethical situations? What experiences do you have of the exercise of power in healthcare? What experiences do you have of the misuse of power or patient violence toward nurses in a psychiatric context? What are your experiences of abuse by carers towards you as a leader? There are several studies and literature on the fact that care can cause additional suffering to a patient—What thoughts does this raise for you in terms of psychiatric care? In your experience, how often and to what extent do situations of unethical behavior towards patients occur in psychiatry? To what extent is unethical behavior brought up for discussion either in the work group or reported to you as a leader? What do you think might prevent staff from discussing or reporting situations of unethical behavior? As a leader, have you noticed patterns where unethical behavior has occurred? Can you share your thoughts on how you think leaders can help prevent patients from being exposed to unethical behavior? What organizational factors could promote an ethical climate and prevent unethical behavior?

An inductive approach was employed to ensure that the data could be analyzed without any preconceptions.^56^ Qualitative content analysis was used to analyze the data, inspired by Graneheim and Lundman.^57^ Data were anonymized so that participants could not be recognized. The data were first read several times, followed by the selection of meaning units considered essential to the aim of the study. The meaning units were then condensed and coded, and the coded meaning units thereafter sorted into subcategories and categories (Table 1).

**Table 1.** An example of the analysis conducted.

Meaning unit	Condensed meaning unit	Code	Subcategory	Category
*We have to be aware of the history that we have, and it is quite horrible*	The history is quite horrible	Horrible history	A dark history	Consequences of unethical conduct and the positive development of psychiatric care
*This is emphasized in particular, this kind of power and arbitrariness in psychiatry here has a pretty dark history*	Psychiatry has a dark history and emphasizes the exercise of power and arbitrariness	Dark history with abuse of power and arbitrariness	A dark history	
*This anti-coercion project…it has brought such softness to this, that the threshold for [using] seclusion is higher*	Project against coercion has brought softness and the threshold for seclusion is higher	Project against coercion has yielded results	Less coercion	
*The restricting has decreased to what is most necessary…in the 90s it was more like the rule than the exception*	Restricting has decreased to what is necessary, previously more the rule than the exception	Restricting has decreased	Less coercion	

### Ethical considerations

Research ethics permission to conduct the study was applied for and received from a Research Ethics Board where the researchers are domiciled. The guidelines on good scientific practice as delineated by the Finnish Advisory Board on Research Integrity TENK were followed during all stages of the study.^58^ Research permission to conduct the study was applied for and granted by relevant individuals at the two organizations included in the study. The participants gave their informed consent.

### Findings

The aim of this study was to explore the phenomenon of unethical conduct in a psychiatric in-patient context from nurse leaders’ perspectives. From the content analysis six main categories were generated: Unethical conduct and violations against patients, Unethical conduct and violations against staff, Unethical conduct and violations by staff against other staff, Unethical conduct and violations against leaders, Reasons underlying unethical conduct, and Consequences of unethical conduct and the positive development of psychiatric care.

### Unethical conduct and violations against patients

According to the participants, unethical conduct with patients can take many different forms and can vary in severity, ranging from failed encounters to a misuse of power. Overall the participants described and mentioned coercion and coercive interventions often, although experiences varied between participants. *Then but it’s really difficult for me to assess how extensive it is, but with us, I definitely cannot say [without lying] that ‘there is no such thing in my wards’ but it’s like that where there are people surely something like this happens* (P3).

The participants perceived that while many different measures could be taken to ensure patient well-being, coercion and/or restricting patients’ actions could occasionally be implemented. Some participants perceived that coercive interventions could be used unquestioningly and automatically. The participants criticized the use of unnecessary coercion with patients yet nonetheless acknowledged that it could occur. *I see in certain situations that a person,* for example, *is isolated in a room or even isolated and mechanically restrained in situations where you in another way should be able to manage this and it escalates the situation* (P6).

The participants experienced that a power imbalance exists in psychiatric contexts, which they attributed not only to the nature of involuntary care but even to staff attitudes. Some participants perceived that the use of power could be beneficent, describing how nurses could protect patients or influence patients toward better well-being. These participants often noted that they perceived that such actions were motivated by a focus on the patient’s best interests.

*This is of course always present for us precisely because we have patients who are easily in the role of the underdog because they are not able to take care of their own rights so well, so we become very aware that our policies and decision-making are not arbitrary or dependent on the individual…* (P5).

Several participants perceived that psychiatric care has historically been characterized by rules through which control over a ward can be attained. Some participants experienced that some rules could be unnecessary because their implementation could lead to a cycle where staff were required to continuously deny patients their requests, that is, staff were required to repeatedly say “no” to patients. The participants could perceive that certain rules on food or tobacco use were paternalistic, routine-focused, or not patient-centered.

*If they watch TV too loudly and we say ‘lower the volume’…[if they] want to make coffee and it’s not mealtime and then we say no then it is like to be sure like this kind of constant no saying and this becomes this kind of certain routine and and I think that you become then a little ja…jaded yourself when you are in this now in the no-saying and and then I think that this exercise of power like very unconsciously becomes becomes a problem* (P6).

### Unethical conduct and violations against staff

The participants perceived that also patients could engage in unethical conduct. The most frequent forms of unethical conduct described by the participants were the verbal abuse of staff, name-calling, and threatening of staff. The participants even experienced that physical violence and aggression could occur on a regular basis and perceived that aggression and violence could be a characteristic of closed psychiatric wards where patients are treated involuntarily. *Well of course there is…that that that the one sad part of our job is that we are often the target of this kind of aggression… subject to aggressive behavior often* (P5).

One participant perceived that patients who sought care voluntarily could have a different relationship with staff while patients who did not seek care voluntarily could more frequently engage in unethical conduct toward staff. The participants nevertheless experienced that staff understand patients’ verbal abuse and even aggression and strive to maintain an empathetic approach when encountering such.

*But with patients we look through our fingers a lot because they are sick and when they are like that it is often so unfortunate that the [disease] may include a lack of impulse control something like when…depression, anxiety can also cause irritability and such short-temperedness* (P3).

However, a few participants even perceived that “a line could be crossed” regarding aggression, i.e., when staff’s well-being and mental health were compromised. The participants experienced that this was related to the severity of patients’ actions or threats.

### Unethical conduct and violations by staff against other staff

The participants perceived that staff could engage in unethical behavior, including the abuse of or violations against other staff. The participants experienced that collegiality could sometimes suffer between wards because an understanding for others’ work could be lacking. Most of the participants perceived that difficulties between staff members could arise because of a lack of interpersonal chemistry or if staff had different levels of knowledge or different approaches to taking initiative. Some participants described how staff with longer experience could be expected to make difficult decisions or give patients unwelcome information, which the participants perceived could lead to a sense of resentfulness.

*Now in all workgroups there are certain carers who are more on and now we do and we fix this we do now we get things done now medicine is needed and these carers can have expressed that they can feel [that it is] very offensive that it always is them who must make these difficult decisions…* (P6).

Several participants perceived that staff exercising power over other staff was unethical and described how there were always certain staff with a “strong character” who could act in a manner that resulted in them being more likely to “get their way”.

*Yeah yes it was in the early stages that if someone is terribly forceful then…[they] will bulldoze it [through]…and it goes kind of at the expense of others kind of like those others don’t get to say anything about it…and then it’s like that unfortunately it just has to be addressed somehow* (P4).

These participants experienced that such behavior could result in resentment between staff and highlighted the importance of staff acting in a responsible manner in the workplace environment so as to ensure everyone’s well-being.

### Unethical conduct and violations against leaders

While encounters with their staff were primarily described as being positive, some participants even described situations that they perceived could be considered ethically questionable, for example, when staff ignored or did not follow leaders’ guidelines or instructions. These participants acknowledged that while the role of leader included receiving criticism and/or negative feedback that they as leaders could sometimes be criticized for matters that they neither could control nor influence, for example, decisions overseen by upper management.

*And often there may be things that are not my decision or in my hands but that in a way I represent the organization in this situation and I have to accept perhaps such frustration that in practice may not be aimed at me but that I nevertheless [represent] that organization so then I accept yes criticism quite willingly but I understand that such output needs to be addressed like that* (P3).

### Reasons underlying unethical conduct

Several participants perceived that staff’s unethical conduct could be caused by various factors, for example, personal factors or concerns. These participants noted that nurses are human beings and thus imperfect: that mistakes can be made when communicating and interacting with others, particularly if one is under pressure. These participants also highlighted professional fatigue as a factor that could underlie unethical conduct. The participants perceived that working long hours and taking care of patients whose recovery takes time could lead to a sense of fatigue and create unethical situations.

*That if the working group so to speak gets tired of a certain patient they no longer can take care of them and that professionalism can suffer as a result, it can then become that the encounter with the patient is no longer friendly, friendly and appropriate, but instead there may be such excesses then whether those choices are made on such an affective basis or whether it’s really like professional treatment and choices* (P5).

One participant experienced that staff conduct could be influenced by staff statements or perceptions about patients that were based on (previous) patient interaction. This participant noted that it was essential that the language and descriptions used by staff about patients be as objective and neutral as possible. For example, the participant perceived that if a patient was characterized or “labeled” in a certain manner staff could employ restrictive measures more often or could engage in unethical conduct, for example, unnecessarily restrictive measures.

*They depend of course a little on the patient…the client also…that they of course clearly these ethical mistakes they surely most often happen when you feel threatened then these ethical mistakes like more emerge…when you feel yourself…when you feel yourself threatened in some way* (P1).

The participants even perceived that a lack of communication could underlie unethical conduct. For example, some participants experienced that patients could experience an encounter with staff as being negative or unethical if there was insufficient communication or if a staff member’s language skills were inadequate. The participants noted that patients’ unethical conduct was to a large extent tolerated by staff and understood to be primarily caused by patients’ ill-health. Nonetheless, a few participants highlighted that “a line could be crossed” and that this was when patient conduct led to the diminishment of staff well-being.

*…I think it’s a pretty [high] threshold for us to tolerate quite a lot and understand that the disease brings with it that kind of maybe inappropriate behavior but then just like that when the patient crosses this line I think that at that point at least the mental stamina is put to the test* (P2).

### Consequences of unethical conduct and the positive development of psychiatric care

Some participants perceived that coercive interventions and in particular mechanical restraint and/or seclusion could cause patients to experience trauma or suffering. *But but of course…coercive measures… ven even though they in* and *of themselves are needed so I think that it that it more or less always causes some form of suffering they can even cause physical suffering physical suffering* (P6).

The participants also experienced that a loss of autonomy or the ability to make decisions could result in patients experiencing suffering. The participants perceived that the restrictions inherent to a psychiatric ward, for example, locked doors and restricted access, could be negative for patients’ recovery.

*Because I think that it is important that they get to process them but there are of course surely people who are involuntarily put into care perhaps [in care] a half year a year and then the day they are released they still don’t understand that they are ill…so that they [are] of course lifelong traumas* (P1).

One participant perceived that patients’ negative care experiences could even impact whether patients would seek further mental health care in the future.

*You have to think about not only the one treatment period and what it causes the patient then but also about….it’s like how it [affects the rest of the patient’s life when it ends] and like how do you seek treatment and also go so far that then they might tell their friends that you shouldn’t go there and you don’t get help there, that in a way it can have such a cumulative effect also that if you don’t see these kinds of abuses and situations like this because it’s really serious because they affect so many people and cause very significant suffering for the patient* (P3).

The participants repeatedly expressed that they perceived that psychiatric care has significantly changed during the past few decades. Some participants described the history of psychiatric care as being dark and coercive and experienced that an awareness of such is essential to inhibit the practice/existence of outdated attitudes in the patient encounter. With some stating that coercive measures have previously been implemented more as a rule than an exception, most participants also perceived that proactive work against the use of coercion in care has begun to yield positive results. The participants perceived that, unlike previously, coercive measures were no longer being used as a form of punishment in psychiatric care.

*We have to be aware of the history we have and it’s pretty horrible like when you think about psychiatric patients they have been treated and oppressed like in the name of treatment and used as free labor [accordingly] and so that it’s like you don’t like think that it hasn’t happened because there’s such a risk that in a way it’s still kind of going to pop up from there if you don’t actively fight back* (P3).

A few participants even perceived that the introduction of a new, younger generation of carers has played a part in improving staff’s ethical conduct and tend to be more positive toward and aware of patients’ rights.

## Discussion

The aim of the study was to explore the phenomenon of unethical conduct in a psychiatric in-patient context from nurse leaders’ perspectives. We found in the studied context that various parties can engage in unethical conduct. Six main categories emerged from the analysis: Unethical conduct and violations against patients, Unethical conduct and violations against staff, Unethical conduct and violations by staff against other staff, Unethical conduct and violations against leaders, Reasons underlying unethical conduct, and Consequences of unethical conduct and the positive development of psychiatric care. To the best of our knowledge, no previous research on nurse leaders’ perspectives on unethical conduct in a psychiatric in-patient setting in which a broader focus has been used exists. Researchers in earlier studies have employed a specific research focus, for example, a focus on coercion or containment methods.^33^

We discovered that unethical conduct against patients can take many different forms and vary in severity, ranging from failed encounters to misuse of power. We discerned that coercion, or violations could be associated with many unethical situations in the study setting. The participants perceived that coercive interventions were necessary in involuntary care but that such were applied in a largely appropriate and minimal manner. We saw that some participants in this study experienced that coercion could occasionally be used with patients in an unquestioning and automatic manner. Several participants also perceived that psychiatric care is characterized by rules that could lead to a cycle where staff could deny patients’ requests and/or repeatedly say “no” to patients. These findings are in line with earlier research where researchers have found that patients can experience that ward rules relevant to mealtimes or the use of tobacco can be unnecessary or rigid.^28,32^ According to the theory of caritative caring safeguarding the patients’ dignity and vulnerability is of key importance to avoid further suffering. The leader sets the ethical tone for the workplace culture. According to earlier research, care can cause further suffering for the patient.^14^ Participants in this study experienced that these types of cultures were harmful but existed due to habit and history.

We also found that unethical conduct and violations against staff could occur. Describing patients’ physical violence and aggression against staff and the verbal abuse of staff, the participants perceived that such behavior could be typical for closed psychiatric wards and involuntary care. Previous research has shown that verbal and physical violence are the most frequent forms of violence against psychiatric staff and that such can be linked to staff’s emotional, psychological, and physical well-being at work.^42^ Promoting staff well-being is key to reducing problems such as burnout, aggression, and coercion for caregivers.^44^ According to the theory of caritative leadership showing staff compassion and empathy will also reflect positively on the patients as well.^53^ In this study, the participants described how they perceived that staff were understanding of patients’ aggression; the participants experienced that such could occur on a regular basis and perceived it as being a characteristic of closed psychiatric wards where patients are treated involuntarily. However, a few participants also highlighted that a line could be crossed when staff’s well-being and mental health suffered. Earlier research has also seen that mental health professionals may not report incidents because they perceive that violations are inherent to their work and that it can be futile to report incidents.^41^

From the findings, we even discerned that unethical conduct and violations by staff against other staff could occur. Most participants perceived that difficulties could arise between individual staff members, which they linked to interpersonal chemistry (or the lack thereof), different levels of knowledge, and different approaches to taking initiative. As seen in previous research, verbal and nonverbal abuse between staff can constitute the most frequent forms of mistreatment that nurses encounter.^45^ Other researchers have also found that younger and more highly educated staff can be more likely than older and less educated staff to report bullying.^47^ We also saw in this study that some participants experienced that staff could exercise power over other staff, which these participants perceived to be unethical. This was seen as, for example, staff members with a strong character, which these participants perceived could lead to certain staff being more likely to get their own way.

Also unethical conduct and violations against leaders were seen. The participants perceived that encounters with other staff were largely positive, but some participants nonetheless described ethically questionable situations. For example, some participants experienced that they could be criticized for things that they had no control over and which they perceived were organizational-related matters, although they could even express understanding for such. Researchers have previously shown that managers can experience fewer violations from staff compared to staff violations against other staff.^49^ Nevertheless, violations against leaders as a phenomenon has not yet been widely explored in research.^48^

From the findings we even discerned various reasons underlying unethical conduct. For example, several participants perceived that personal factors or concerns could underlie staff’s unethical conduct. These participants experienced that staff errors in communicating and interacting with patients could occur, particularly when staff were under pressure, and even highlighted the role of professional fatigue in unethical conduct. In previous research, researchers have found that psychiatric patients can experience that nursing staff can act in an unprofessional manner, which may be linked to nurses’ burnout and exhaustion.^59^ Other researchers have seen that patients’ challenging behavior can be associated with neglected needs, harmful nursing, and coercive nursing cultures in which inappropriate medication or inappropriate coercive interventions are used.^21^ Bondas^53^ states that health care employees, stakeholders, and the leader all share the same goal: ministering to the patients. In this relationship openness, availability and dialogue are necessary elements. Bondas claims that when there is no open discussion and value conflicts are not brought to light the core idea could be lost and violations could take place in the care culture. In this study, one participant also experienced that earlier staff statements or perceptions about patients could impact staff conduct, and this participant perceived that restrictive measures could be used if patients were characterized or labeled in a certain way. Even other researchers have previously found that in psychiatric inpatient care some staff can distance themselves from patients they find particularly challenging.^32^

We discerned from the findings various consequences of unethical conduct and the positive development of psychiatric care. Various participants perceived that coercive interventions (particularly restraint and/or seclusion) or the loss of autonomy inherent to a psychiatric ward could cause patients trauma or suffering. Also other researchers have seen that patients can experience coercive measures as being more humane if they are not left alone and/or if staff members are present.^21^ Patients can experience suffering related to care if they do not feel seen or valued as a human being,^14^ and involuntary care and coercion, through which autonomy is inherently limited, always causes a level of suffering.^5^ We also saw in this study that one participant perceived that negative care experiences could impact whether patients would in the future seek mental health care, which is in line with previous research.^23^ We moreover found that psychiatric care has evolved in a more ethical direction during the past few decades: toward less coercion and an increased awareness of patients’ rights. While some participants noted that psychiatric care has had a dark and coercive past, most participants nonetheless perceived that proactive work against coercion in care and attitudes among the new younger generation of carers have contributed to the positive changes seen today.

## Methodological considerations

One limitation might be that perceptions of what constitutes unethical conduct may differ, relevant to each ward’s ethical climate and values. It is possible that those nurse leaders who elected to participate in this study are more interested in ethics and creating an ethical atmosphere than their peers, which can have impacted the results. Also, a focus on administrative work is inherent to the role of nurse leader, thus participants are less involved in direct clinical work. One strength is that all of the participants had lengthy experience of clinical nursing work. The small sample size might be a limitation. A strike among healthcare workers in Finland began during participant recruitment (spring 2022), resulting in one potential participant withdrawing from participation in the study.

## Conclusion

From the findings, we discerned new knowledge on the phenomenon of unethical conduct in psychiatric care from a leadership point of view. Unethical conduct was seen to be a multifaceted phenomenon, and patients and staff alike can both experience and engage in various forms of unethical conduct. Unethical conduct against patients was linked to power imbalance (nature of involuntary care and staff attitudes) and a focus on rules based in historical precedent (paternalistic, routine-focused, and not patient-centered). Unethical conduct against staff was linked to the nature of involuntary care and patient ill-health. Unethical conduct by staff against other staff was linked to a lack of understanding for others’ work, interpersonal chemistry, (length of) work experience, and staff character. Unethical conduct against leaders was linked to leaders being perceived as the organization. The findings can contribute to improved and broader understanding of unethical conduct as a phenomenon, including that various parties can engage in unethical conduct in a psychiatric setting. This paper provides new insight by explaining unethical conduct as a multifaceted phenomenon, where all parties—patients, staff, and leaders—can become vulnerable to unethical conduct, whether intentional or not. It examines these dynamics in a psychiatric care context and broadens the concept of ethics in mental health. Furthermore, the study highlights ethical conduct and workplace culture, with a particular focus on leadership.

## Data Availability

Due to the sensitive nature of the research conducted, the supporting data are not available.
